# Lobenzarit disodium inhibits the constitutive NO–cGMP metabolic pathways. Possible involvement as an immunomodulatory drug

**DOI:** 10.1155/S0962935195000597

**Published:** 1995-09

**Authors:** J. Padrón, A. Rojas, L. Glaría, L. Caveda, R. Delgado, M. Torres, O. Martínez, E. López, A. Beltrán, M. Palacios

**Affiliations:** 1 Center of Pharmaceutical Chemistry Department of Pharmacology and Toxicology Havana City POB 6990 Cuba

## Abstract

Lobenzarit disodtulIl (CCA) is a novel immunomodulatory drug useful in the treatment of chronic inflammations. Its principal mechanism of action seems to be through enhancing the T suppressor/T helper lymphocyte ratio. However, the molecular basis for these actions remains unclear. In this study it was found that CCA inhibits the production of guanosine 3',5'-cyclic monophosphate almost completely when present in concentrations of 1 mM. Further results demonstrated that such inhibition could also be explained by interference in constitutive nitric oxide generation. In addition to previous findings, more insight into the molecular mechanism of action of CCA is provided.


Mediators of Inflammation 4, 364–367 (1995)

### Lobenzarit disodium inhibits the constitutive NO–cGMP metabolic pathways. Possible involvement as an immunomodulatory drug

J. Padrón,[CA] A. Rojas, L. Glaría, L. Caveda, R. Delgado, M. Torres, O. Martínez, E. López, A. Beltrán and M. Palacios

Center of Pharmaceutical Chemistry, Department of Pharmacology and Toxicology, POB 6990, Havana City, Cuba

[CA]Corresponding Author

**LOBENZARIT disodium (CCA) is a novel immunomodulatory drug useful in the treatment of chronic inflammations. Its principal mechanism of action seems to be through enhancing the T suppressor/T helper lymphocyte ratio. However, the molecular basis for these actions remains unclear. In this study it was found that CCA inhibits the production of guanosine 3′,5′-cyclic monophosphate almost completely when present in concentrations of 1 mM. Further results demonstrated that such inhibition could also be explained by interference in constitutive nitric oxide generation. In addition to previous findings, more insight into the molecular mechanism of action of CCA is provided.**

**Key words:** Anti-inflammatory action, cGMP, Chronic inflammation, Immunomodulator, Lobenzarit disodium, Molecular mechanism, Nitric oxide.

### Introduction

*N*-(2-carboxyl phenyl)-4-chloro anthranilic acid disodium salt (CCA), known as lobenzarit disodium, is a novel immunomodulatory drug useless in the treatment of acute inflammation[1] but experimentally very useful in the treatment of chronic inflammatory auto-immune diseases such as rheumatoid arthritis[2] and diabetes.[3] Its mechanism of action may be related to its capacity to enhance the T suppressor/T helper lymphocyte ratio.[4] However, from a molecular point of view the nature of the effect remains unclear.

CCA is a radical scavenging molecule derived from anthranilic acid[5] which as far as is known shares structural features with known inhibitors of the guanylate cyclase pathway, such as chlorpromazine and methylene blue. Considering the crucial role of cyclic nucleotides in many of the activation processes of the immune system[6] we decided to investigate the possible effect of CCA upon the generation of guanosine 3′,5′-cyclic monophosphate (cGMP) and the closely related nitric oxide (NO) metabolic pathway.

Two major nitric oxide synthases (NOS) have been reported: the inducible pathway (iNOS), that is mainly dependent on inflammatory stimuli,[7] and the constitutive pathway (cNOS), that is controlled by calmodulin and cytosolic calcium levels.[8,9] Both enzymes are used by L-arginine in the presence of molecular oxygen to produce L-citrulline and NO,[10] although the distinguishable kinetic effects of the cNOS[11] enable it to mediate in the generation of cGMP.[9]

cGMP, in particular, is known to be responsible for many immune inflammatory processes including macrophage activation,[12] lymphocyte proliferation,[13] vascular smooth muscle relaxation,[14] mast cell degranulation,[15] chemotaxis[16] and platelet aggregation,[17] and adhesion to endothelium.[18] Therefore we investigated the effect of CCA upon the constitutive NO–cGMP metabolic pathways, in order to gain more insight into the pharmacodynamics of CCA, which might explain its therapeutic proficiency in the treatment of chronic inflammatory diseases.

### Materials and Methods

*Chemicals:* CCA was synthesized by Dr R. Pellón and colleagues at the Center of Pharmaceutical Chemistry in Havana, Cuba.[19] The radioimmunoassay kit for cGMP determinations and $^{14}$C-labelled L-arginine were obtained from Amersham International. The specific NOS inhibitor L-$N^G$-monomethyl arginine (L-NMMA) was a kind of gift from Dr S. Moncada at Wellcome Research Laboratories. All other chemicals were purchased from Sigma.

*Cytosol preparation:* Brains of male Sprague–Dawley rats weighing 180–200 g were used as the best source for the conversion of cytosol into both guanylate cyclase and cNOS. As described previously,[9] after decapitation rat forebrains were extracted and washed in ice-cold sucrose buffer (sucrose 0.32 M, HEPES 10 mM, DL-dithiothreitol

© 1995 Rapid Science Publishers

1 mM, pH 7.4), and thereafter homogenized in an appropriate buffer at pH 7.4 containing Tris-HCl (50 mM), EDTA (0.1 mM), EGTA (0.1 mM), dithiothreitol (0.5 mM), phenylmethylsulphonyl fluoride (1 mM), pepstatin A (1 μM) and leupeptin (2 μM). Once extracted, the cytosol samples were kept at 0–4°C for no longer than 15 min before assay of cGMP and L-citrulline production.

*Biochemical assays:*

cGMP assay. According to previous reports,[9] 150 μl of cytosol were mixed with 50 μl of a buffer containing Tris (25 mM), $MgCl_2$ (5 mM), L-arginine (100 μM), $CaCl_2$ (2 mM), 3-isobutyl-1-methyl xanthine (1 mM), GTP (5 mM) and CCA (1000, 100, 10 or 0 μM). Total cGMP level in each fraction was quantified after 10 min of incubation at 37°C by using RIA following the manufacturer's instructions.

[$^{14}$C]-L-Citrulline assay. As described elsewhere,[20] 25 μl of cytosol were mixed with 100 μl of an appropriate buffer at pH 7.4 containing [$^{14}$C]-L-arginine, Tris-HCl (50 mM), L-arginine (100 μM), NADPH (100 μM), $CaCl_2$ (2 mM) and CCA (3000, 300, 30, 3 or 0 μM). Final amount of [$^{14}$C]-citrulline generated after 10 min of incubation at 37°C was determined in each fraction by liquid scintillation counting coupled with a set of columns for ionic exchanging chromatography (Biorack).

*Statistical analysis:* All values were expressed as mean ± standard deviation. The number of experiments ($n$) is also shown for each case and was never less than three replicate experiments. Significant differences between the control group and the test groups was assessed using Student's $t$-test comparison; $p$ values less than 0.05 or 0.01 were considered significantly different.

### Results

After 10 min of cytosol incubation at 37°C with the appropriate buffer, CCA spontaneously inhibited the generation of cGMP (Table 1). This inhibition clearly shows a concentration-dependent shape in the range between 0.01 and 1 mM of CCA. A 100% inhibition of the total amount of cGMP generated was reached at 1 mM of CCA. To assess the real amount of cGMP produced in 10 min, in each case the basal cGMP level (background) was subtracted.

Further results demonstrated that CCA is also capable of inhibiting, in a concentration-dependent manner, the constitutive generation of L-citrulline after 10 min of cytosol incubation at 37°C with the appropriate buffer (Table 2). This finding indicates an inhibitory activity in the NOS metabolic pathway which reaches a maximum of more than 70% of the inhibition achieved by the specific antagonist L-NMMA.

It should be noted also that CCA when present at 3 mM scarcely reaches the 70% of the cNOS

Table 1. Inhibitory effect of CCA upon the guanylate cyclase activation pathway

| | Background (at time 0) | +CCA (0 mM) | +CCA (0.01 mM) | +CCA (0.1 mM) | +CCA (1 mM) | +L-NMMA (0.05 mM) |
|---|---|---|---|---|---|---|
| cGMP (pmol) | 2.7 ± 0.43 | 12.4 ± 1.11 | 7.6 ± 1.78* | 3.4 ± 0.26† | 2.0 ± 0.02† | 4.7 ± 0.80† |
| Inhibition (%) | - | - | 50.3 | 91.7 | 106.6 | 78.6 |
| Replicates | $n$=6 | $n$=5 | $n$=5 | $n$=3 | $n$=3 | $n$=6 |

*$p < 0.05$ and †$p < 0.01$ when compared with the production of cGMP in the control group (+CCA 0 mM). To assess the real amount of cGMP generated in 10 min, the level of cGMP present at time zero (background), was for each case subtracted. Percentages of inhibition are calculated by comparison with the total cGMP generated after 10 min in the absence of CCA. L-NMMA was used as an additional control because of the involvement of the cNOS metabolic pathway in the generation of cGMP.

Table 2. Inhibitory effect of CCA upon the cNOS activation pathway

| | +CCA (0 mM) | +CCA (0.003 mM) | +CCA (0.03 mM) | +CCA (0.3 mM) | +CCA (3 mM) |
|---|---|---|---|---|---|
| NOS activity (nmol/mg/min) | 137.7 ± 3.1 | 133.0 ± 2.5 | 112.5 ± 1.8* | 104.4 ± 21.2* | 38.1 ± 7.4* |
| Inhibition (%) | 0 | 3.4 | 18.3 | 24.2 | 72.3 |
| Replicates | $n$=6 | $n$=4 | $n$=4 | $n$=4 | $n$=3 |

*$p < 0.01$ when compared with the control group (+CCA 0 mM).

inhibition achieved by L-NMMA at 0.05 mM, whereas CCA at 1 mM exceeds the inhibitory action of L-NMMA at 0.05 mM in cGMP generation.

### Discussion

Considering that the inhibition of the guanylate cyclase system by CCA proved to be sufficient to reduce cGMP levels by 50% even at 0.01 mM, this action will probably have biological significance in terms of its molecular pharmacodynamics when *in vivo* conditions are considered.

Curiously, in our system L-NMMA (0.05 mM) does not abrogate the production of cGMP to the same extent as is observed for NO generation. This could be either because there is another NO-independent mechanism for guanylate cyclase stimulation[21] or because L-NMMA (0.05 mM) actually fails to affect NO generation, leaving a low level of cNOS activity. In any case, the results showing the inhibitory action of CCA upon the generation of NO are consistent and could provide an explanation for the inhibition of cGMP production achieved by CCA. However, for the cNOS system the inhibitory potential of CCA at 3 mM seems to be slightly lower, reaching 70% of the inhibition caused by L-NMMA at 0.05 mM.

Regarding the fact that CCA (1 mM) is able to inhibit cGMP generation to a greater extent than is seen by L-NMMA (0.05 mM), and furthermore CCA (3 mM) has a lower inhibitory action than L-NMMA (0.05 mM) upon the generation of NO, it is possible that in addition to cNOS inhibition, CCA has another inhibitory effect on the NO–cGMP metabolic pathway. Such additional inhibition (25%) has indeed been observed when an exogenous NO-releasing molecule (SNAP) was used as a cNOS-independent mechanism for guanylate cyclase stimulation (data not shown). That could be either due to a NO scavenger activity (depending on its nitrosable diphenylamine nitrogen) or to a direct inhibition of the guanylate cyclase enzyme (by comparison of its structural similarities with the known guanylate cyclase inhibitor methylene blue).

It could be important also to elucidate the mechanism by which CCA inhibits cNOS activity. The calcium-calmodulin dependence of the cNOS,[8] the strong calcium chelating properties of CCA (Dr R. Pellón, personal communication, and Reference 19) and the structural resemblance of CCA with chlorpromazine (a calmodulin antagonist that recently has been reported to be an inhibitor for the activation of brain cNOS and a suppressor for LPS-induction of iNOS in the lung[22]) have given weight to the idea that the calcium-calmodulin system is the predominant system involved in such inhibition.

In summary, considering the role of cNOS for mediating the induction of the iNOS[22,23] in addition to the capacity for high NO levels to cause tissue damage[24] and to suppress T helper type 1 cells[25] (which often work like 'T suppressor cells' for antibody production), it is possible to suggest that most of the therapeutic effects of CCA are to a great extent due to its capacity to inhibit the NO–cGMP metabolic pathway, which is known to play a critical role in arthritis[26] and diabetes.[27] Additionally, since most of the cNOS share their calcium-calmodulin dependence,[28] there are many other potential effects of CCA that should be investigated in future. In fact, there are several actions of CCA that potentially could be explained by such effects. The results of the present work may indicate a new course for investigations of the pharmacodynamics of lobenzarit disodium that may result in the search for novel strategies for the therapy of chronic inflammatory auto-immune diseases.

### References

1. Tanemura M, Nakano T, Ohsugi Y, *et al.* Effect of an immunomodulator 'lobenzarit disodium (CCA)' in various models of inflammation. *Jap J Inflammation* 1984; **4:** 239–242.
2. Itokazu M, Tanaka S. Anti-arthritic effect of a newly synthesized immunomodulating drug: lobenzarit. *Jap J Inflammation* 1983; **3:** 244–247.
3. Iwakiri R, Nagafuchi S. Inhibition of streptozocin-induced insulitis and diabetes with lobenzarit in CD-1 mice. *Diabetes* 1989; **38:** 558–561.
4. Matsuno H, Matsushita I, Kadowaki KM, *et al.* Effects of lobenzarit disodium on lymphocyte subsets related to the onset of collagen-induced arthritis. *Int J Immunotherapy* 1992; **VIII(2):** 67–75.
5. Cynshi O, Saitoh M, Cynshi F, Tanemura M, Hata S, Nakano M. Anti-oxidative profile of lobenzarit disodium. *Biochem Pharmacol* 1990; **39:** 2117–2122.
6. Watson J. The influence of intracellular levels of cyclic nucleotides on cell proliferation and the induction of antibody synthesis. *J Exp Med* 1975; **141:** 97–111.
7. Stuehr DJ, Cho HJ, Kwon NS, Weise M, Nathan CF. Purification and characterization of the cytokine-induced macrophage nitric oxide synthase: an FAD- and FMN-containing flavoprotein. *Proc Natl Acad Sci USA* 1991; **88:** 7773–7777.
8. Bredt DS, Snyder SH. Isolation of nitric oxide synthase, a calmodulin requiring enzyme. *Proc Natl Acad Sci USA* 1990; **87:** 682–685.
9. Knowles R, Palacios M, Palmer R, Moncada S. Formation of nitric oxide from L-arginine in the central nervous system: a transduction mechanism for stimulation of soluble guanylate cyclase. *Proc Natl Acad Sci USA* 1989; **86:** 5159–5162.
10. Leone A, Palmer R, Knowles R, Francis P, Ashton D, Moncada S. Constitutive and inducible nitric oxide synthase incorporate molecular oxygen into both nitric oxide and citrulline. *J Biol Chem* 1991; **226:** 23790–23795.
11. Knowles R, Palacios M, Palmer R, Moncada S. Kinetic characteristics of nitric synthase from rat brain. *Biochem J* 1990; **269:** 207–210.
12. Bromberg Y, Pigk E. Cyclic GMP metabolism in macrophages. *Cell Immunol* 1980; **52:** 73–83.
13. Hadden J, Hadden E, Haddeox M, Goldberg N. Guanosine 3',5'-cyclic monophosphate: a possible intracellular mediator of mitogenic influences in lymphocytes. *Proc Natl Acad Sci USA* 1972; **69:** 3024–3027.
14. Gruetter C, Barry B, McNamara D, Gruetter D, Kadowitz P, Ignarro L. Relaxation of bovine coronary artery and activation of coronary arterial guanylate cyclase by nitric oxide, nitroprusside, and a carcinogenic nitrosamine. *J Cyclic Nucleotide Res* 1979; **5:** 211–224.
15. Salvemini D, Masini E, Anggard E, Mannaioni P, Vane J. Synthesis of a nitric oxide-like factor from L-arginine by rat serosal mast cells: stimulation of guanylate cyclase and inhibition of platelet aggregation. *Biochem Biophys Res Commun* 1990; **169:** 596–601.

16. Kaplan S, Billiar T, Curran R, Zdziarski U, Simmons R, Basford R. Inhibition of chemotaxis with $N^G$-monomethyl-L-arginine: a role for cyclic GMP. *Blood* 1989; **74:** 1885–1887.
17. Mellion B, Ignarro L, Ohlstein E, Pontecorvo E, Hyman A, Kadowitz P. Evidence for the inhibitory role of guanosine-3′,5′-monophosphate in ADP-induced human platelet aggregation in the presence of NO and related vasodilators. *Blood* 1981; **57:** 946–955.
18. Radomski M, Palmer R, Moncada S. The role of nitric oxide and cGMP in platelet adhesion to vascular endothelium. *Biochem Biophys Res Commun* 1987; **148:** 1482–1489.
19. Pellóon R, Millian V, Carrasco R. Procedure for the synthesis of substituted *N*-phenylanthranilic acids. *1992 Cuban patent No. 22105.*
20. Palacios M, Knowles R, Palmer R, Moncada S. Nitric oxide from L-arginine stimulates the soluble guanylate cyclase in adrenal glands. *Biochem Biophys Res Commun* 1989; **165:** 802–809.
21. Beasley D, McGuiggin M. Interleukin 1 activates soluble guanylate cyclase in a human vascular smooth muscle through a novel nitric oxide-independent pathway. *J Exp Med* 1994; **179:** 71–80.
22. Palacios M, Padron J, Glaria L, *et al.* Chlorpromazine inhibits both the constitutive nitric oxide synthase and the induction of nitric oxide synthase after LPS challenge. *Biochem Biophys Res Commun* 1993; **196:** 280–286.
23. Glaría L, Delgado R, Rojas A, *et al.* Role of brain nitric oxide synthase in the endotoxic shock. In: Moncada S, Feelish M, Busse R, Higgs EA. eds. *Biology of Nitric Oxide. Volume 3: Clinical and Physiological Aspects.* London: Portland Press Ltd, 1994; 302–305.
24. Hibbs J, Vavrin Z, Taintor R. L-arginine is required for the expression of the activated macrophage effector mechanism causing selective metabolic inhibition of target cells. *J Immunol* 1987; **138:** 550–565.
25. Taylor-Robinson A, Liew F, Severn A, *et al.* Regulation of the immune response by NO differentially produced by T helper type 1 and T helper type 2 cells. *Eur J Immunol* 1994; **24:** 980–984.
26. Ialenti A, Moncada S, Di Rosa M. Modulation of adjuvant arthritis by endogenous nitric oxide. *Br J Pharmacol* 1993; **110:** 701–706.
27. Corbett J, Wang J, Hughes J, *et al.* Nitric oxide and cGMP formation induced by interleukin 1β in islets of Langerhans. *Biochem J* 1992; **287:** 229–235.
28. Sievert P, Wright S, Larric J. Examination of brain type of nitric oxide synthase expression in non-neural tissues by RT-PCR. *Clontechniques* 1992; **7:** 1–3.

**Received 9 May 1995;**
**accepted in revised form 13 July 1995**
